# Cultural Safety as an Outcome of a Dynamic Relational Process: The Experience of Inuit in a Mainstream Residential Addiction Rehabilitation Centre in Southern Canada

**DOI:** 10.1177/10497323221087540

**Published:** 2022-03-29

**Authors:** Julie Lauzière, Christopher Fletcher, Isabelle Gaboury

**Affiliations:** 1 Département de médecine de famille et de médecine d’urgence, 198734 Université de Sherbrooke, Longueuil, QC, Canada; 2 Département de médecine sociale et préventive, 12369 Université Laval, Quebec, QC, Canada

**Keywords:** Indigenous peoples, Inuit, experiences, cultural safety, trust, health services, Canada, qualitative research

## Abstract

Few addiction treatment options are available in Arctic Canada, leading many Inuit to seek treatment programs in southern cities. We conducted a case study to understand what contributes to a culturally safe experience for Inuit in a mainstream addiction rehabilitation centre in Southern Canada. We carried out more than 700 hours of participant observation, in addition to semi-structured interviews and member-checking activities with 20 Inuit residents, 18 staff and four managers. Data were analysed using an inductive interpretative process. Throughout their journey in the program, Inuit navigated through contrasting situations and feelings that we grouped under six broad themes: having Inuit peers, having limitations imposed on one’s ways of being and doing, facing ignorance and misperceptions, having conversations and dialogue, facing language barriers and being in a supportive and caring environment. This study highlights how cultural safety varies according to people, context and time, and relates to developing trustful relationships.

## Introduction

Significant health disparities exist between Indigenous and non-Indigenous populations worldwide (Gracey & King, 2009), with comparatively high rates of substance use and their related impacts being important dimensions of these disparities (Gone & Trimble, 2012). Substance use is a major concern for Inuit communities in Canada (Bélanger et al., 2020; Inuit Tapiriit Kanatami, 2014; Laneuville, 2015). It is both a cause of social difficulties and a consequence of individual and collective experiences of disempowerment and suffering (Brady, 1995; Brunelle et al., 2009; Chansonneuve, 2007; Inuit Tapiriit Kanatami, 2014). For Inuit, these experiences are related to historical and contemporary events that include instances of forced relocation and coercive policy to encourage resettlement, epidemics and mass medical evacuations to sanatoriums in southern parts of the country, the slaughter of sled dogs and the separation of families as a result of various government policies such as the residential school system (Muckle et al., 2020; Truth and Reconciliation Commission, 2015; Viens, 2019). This results in high rates of individual traumatic lifetime experiences including sexual, physical and emotional abuse; loss of loved ones to suicide; as well as material deprivation and enforced cultural dislocation through colonial institutions. In such a context, the provision of health and social services can be viewed as part of larger colonial processes that have marginalized people from their traditional homelands and livelihoods (O’Neil, 1986).

Responses to addictions are largely provided through systems and mechanisms that can reinforce disparities. Health systems are social spaces in which power relations intersect with social categories such as race, class and gender (Tang et al., 2015). It is generally accepted that a failure to take into account the role of historical experiences, social distance and culture in the delivery of care impedes access for Indigenous populations, thus reinforcing the inequities inherent to health systems (Horrill et al., 2018; King et al., 2009). For Inuit seeking help to reduce or stop using alcohol or drugs, there are limited treatment options that are cognizant of Inuit history, culture, and language and their impacts on addiction behaviours, causes and healing modalities. This situation combined with other considerations such as concerns over confidentiality or court requirements leave many Inuit with little choice but to attend mainstream or First Nations addiction rehabilitation programs in southern cities where linguistic, cultural and historical knowledge and social experiences are not shared by service providers and Inuit service users (Laneuville, 2015; Viens, 2019). Little is known about the experiences of Inuit in such settings. Nevertheless, an extensive literature documents the geographic, social, economic, cultural, linguistic, jurisdictional and institutional barriers that Indigenous people face in accessing care and their often-negative experiences in urban healthcare settings (e.g. Allan & Smylie, 2015; Browne et al., 2011; Goodman et al., 2017; Hole et al., 2015; National Collaborating Centre for Indigenous Health, 2019; Vang et al., 2018).

### Cultural Safety

The recognition of pervasive inequity and discrimination led Maori nurses in New Zealand to propose ‘Cultural Safety’ as a guiding principle and an analytical perspective for considering the influence of the broader historical, sociocultural, political and economic context in the delivery of health services and the creation of health disparities (Ramsden, 2002). Cultural safety emphasizes the culture of health care as a ‘site for transformation’ to address power imbalances, discrimination and the persistent impacts of historical injustices on health and healthcare (Browne et al., 2016, p. 5). It has been examined in the literature as a process and an outcome. As a process of considering culture in care, cultural safety implies critical reflection from healthcare providers and organizations to recognize and question their own biases and the balance of power in care relationships, as well as to implement consequent professional, organizational and system transformations to attend to power differentials (Blanchet-Cohen & Richardson/Kinewesquao, 2017; Brascoupé & Waters, 2009). As an outcome determined by recipients or care, cultural safety refers to what people feel or experience when their cultural identity and worldview are acknowledged and respected through inclusive relationships, sincere commitment, dialogue, equitable partnership and shared decision-making (Blanchet Garneau & Pepin, 2012; National Aboriginal Health Organization, 2008).

To date, studies that have used cultural safety as an analytical lens examined experience of care cross-sectionally (e.g. Browne & Fiske, 2001; Cameron et al., 2014; Hole et al., 2015). We know little about how cultural safety actualizes and/or changes over time in the context of sustained long-term care relationships. In this article, we examined experiences of Inuit in a mainstream addiction rehabilitation centre to identify contexts and practices that promote or hinder cultural safety for Inuit in residential programs and how all of this unfolds over time.

## Methods

### Study Design and Setting

This article is part of a larger project which aims to explore the contribution of cultural safety to identify ways to improve access and services offered to Inuit in mainstream residential addiction rehabilitation programs (Lauzière, 2021; Lauzière et al., 2021). We conducted a qualitative, instrumental case study to allow for a more complete and nuanced understanding of cultural safety as a socially constructed phenomenon (Abma & Stake, 2014; Stake, 1995). Because cultural safety is determined by the individuals or groups likely to experience insecurity (Blanchet Garneau & Pepin, 2012; Ramsden, 2002), we purposively selected an addiction treatment centre that serves a large number of Inuit to maximize what we could learn from the case (Stake, 1995).

The study was conducted in an addiction rehabilitation centre with residential programs in the southern part of the province of Quebec, Canada. As a private institution with agreements with the Quebec Ministry of Health and Social Services, the centre offered voluntary mainstream outpatient and residential addiction rehabilitation programs, as well as aftercare and continuing care programs, social and employment reintegration services, and family services. It served different population groups: adolescents, adults, pregnant women and mothers with young children, adults with mental health issues and individuals referred by the justice system. Over time, the centre had worked with individuals from different ethno-cultural backgrounds including Indigenous peoples, with Inuit representing less than five percent of its program users.

Within a therapeutic community approach (National Institute on Drug Abuse, 2015), program users supported each other in their recovery, in the development of social competencies to better manage challenging situations in their everyday life without resorting to alcohol or drugs. All program users also worked with a case worker on individualized care plans. The length of residential programs was up to 6–8 months, depending on individuals’ needs, motivation and readiness for treatment. New residents were admitted weekly and progressed in gender-specific five-phase programs. Programs were highly structured both in terms of daily schedules and internal social organization according to the residents’ progress. Programs were provided in French and English in a complex linguistic dynamic where staff and residents with varying language competence interact daily.

Within special projects intended for them, Inuit in the residential programs (‘Inuit residents’) gathered approximately once a month in one location outside the centre’s premises, where they could eat traditional foods; the group also met with visiting Inuit Elders who came quarterly to share their life experiences with Inuit who were in institutions in the South (treatment or detention centres). Starting in 2018, a few staff had also been visiting selected villages in northern Quebec, approximately monthly, to offer aftercare support to former Inuit residents.

### Data Collection and Participants

Data collection and analysis were informed by ethnographic and creative research methods (Creswell, 2013; Kara, 2015). The work was performed by a team of non-Indigenous researchers consisting of one female PhD candidate with work experiences in community/public nutrition and Inuit health and two university professors with combined experiences in healthcare organization; qualitative methods; and Inuit culture, health and healing. Ethical approval of the study was received from the local institutional ethics committee (CIUSSS de l’Estrie–CHUS, Sherbrooke, QC, Canada). Participants provided written or oral informed consent upon their preference.

**Figure 1.**
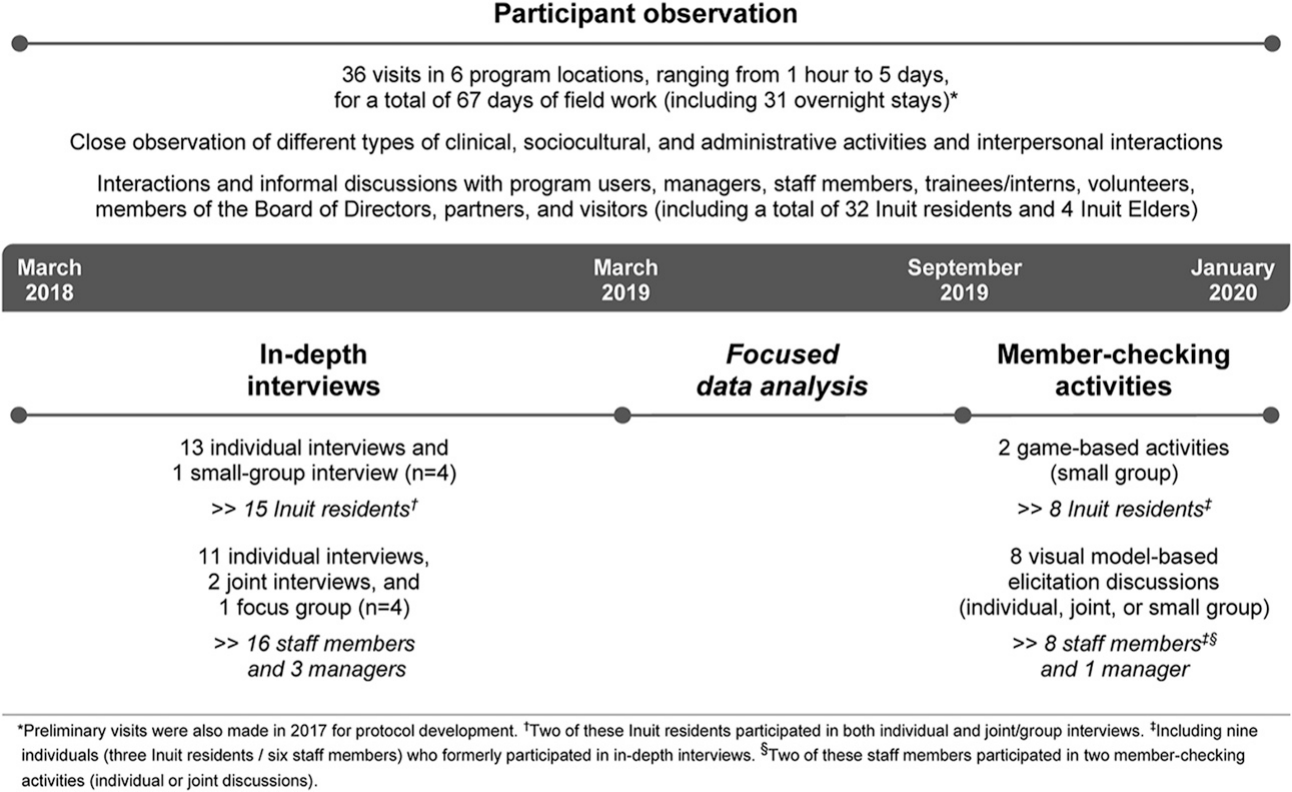
Data collection.

Over a 2-year period, the PhD candidate (Julie Lauzière) collected data using participant observation, document review and in-depth interviews, followed by member-checking activities. Figure 1 summarizes the data collection methods and participants.

### Participant Observation

Participant observation helped the PhD candidate to become familiar with the centre’s activities, functioning and context of intervention, and to establish rapport with its various actors (Bogdewic, 1999; Stake, 1995). At the beginning of each visit, she introduced herself as a university student to everyone she met and made sure to answer any questions they might have about her presence and/or the research project. During visits, she shared the daily lives of Inuit women residents, allowing for close observation of a variety of activities and interpersonal interactions. These were purposively selected and included different types of group meetings, sports and art activities, as well as tasks associated with managing the therapeutic community, preparing meals and maintaining the facilities. On occasion, she observed case conferences or individual interventions with Inuit residents, with their consent. She also spent time interacting with staff and managers during each visit and participated in a few team meetings. Observations were recorded as field notes; covering contexts, activities, actors, interactions, resources and informal discussions with various actors. Selected documents, including the centre’s website, annual reports and program tools were also examined to contextualize observational and interview data.

### In-Depth Interviews

Inuit residents in three of the centre’s adult programs as well as staff and managers were purposively invited to participate in in-depth interviews. Invitations to Inuit residents were made to maximize diversity based on age, sex/gender and life experiences. Other participants were selected based on their role and the intensity of their contact with Inuit residents. The research team developed semi-structured, open-ended interview guides based on the literature and preliminary understanding of the therapeutic context. The interview guides were discussed with an Inuit community organization director before pretesting with one Inuit resident from the case who later agreed for her interview to be retained for analysis. Interview guides evolved over time based on collected data.

All interviews were conducted by the PhD candidate in English or French, according to each participant’s preference, in closed rooms on the centre’s premises. In-depth interviews lasted 35–110 minutes with Inuit participants and 25–200 minutes with staff participants. Interviews were audio-recorded and transcribed verbatim. For participants who were uncomfortable with audio-recording (*n* = 3), minimal notes were taken during the interview and expanded thereafter.

### Data Analysis

We conducted an interpretive thematic analysis (Thorne, 2016) iteratively using a combination of editorial and immersion-crystallization approaches to structuring the analytical process (Miller & Crabtree, 1999). The perspectives of Inuit and staff participants were analysed separately before being contrasted. The PhD candidate immersed herself in data by listening to audio recordings and reading interview transcripts and field notes. Together with a co-researcher (Isabelle Gaboury), she (re)read and examined a selection of transcripts covering each participant category and interview type to identify emergent themes and to form an initial, broad-based coding scheme. The PhD candidate then coded the transcripts using NVivo and refined the initial coding categories. Additional codes were applied to incorporate the temporal and emotional dimensions of the participants’ experiences (Saldaña, 2016). Data were mapped and carefully examined for relationships, patterns and possible connections to existing literature. Observational and interview data were combined to enhance validity of findings through triangulation (Stake, 1995). Team discussions took place throughout the research process to further enhance the trustworthiness of the study findings and interpretations.

### Member-Checking Activities

Following preliminary data analysis, the PhD candidate developed a variation of the ‘Snakes and Ladders’ board game to share and discuss her main observations and preliminary interpretations with two groups of Inuit women residents (Neuwelt & Kearns, 2017). Separate meetings were organized with selected staff members and a manager in which visual models were used to elicit similar discussions (Glegg, 2019). Additional nuances or examples given by the participants during these activities were included in the final analysis.

### Participant Characteristics

Over the course of the study, individuals of different backgrounds and roles were met, including 32 Inuit residents (28 women and four men) from five of the centre’s residential programs. A total of 20 Inuit residents (19 women and one man) aged 18–61 years participated in interviews and/or member-checking activities. All were originally from the Inuit Nunangat and two of them had been living in southern Quebec for over 2 years. Staff participants included 18 clinical/specialized staff and four clinical/administrative managers (15 women and seven men) aged 24–67 years. None of them self-identified as Indigenous. Two-thirds had a lifetime’s experience of problematic alcohol and/or drug use, with some of them being former residents of the centre’s programs. Staff participants had from 1 to 33 years of experience in the field of addiction treatment, including 6 months to 23 years in their current job.

## Findings

Through their narratives, Inuit participants spoke about their experience of navigating between contrasting yet interrelated situations and feelings throughout their program. We grouped these into six broad themes based on the interpretative analysis: having Inuit peers, having limitations imposed on one’s ways of being and doing, facing ignorance and misperceptions, having conversations and dialogue, facing language barriers and being in a supportive and caring environment. After giving an overview of Inuit participants’ journey into the program, we provide a detailed and nuanced description of when and how these interrelated elements manifested and operated in the program to either foster or hinder their cultural safety. The themes are presented in a way that is meant to emphasize the dynamic interplay between them. Data from staff interviews and participant observation are brought into the narrative presented here, when they shed additional light on the context or relational dynamics in the program. All quotes are from Inuit residents, identified by pseudonyms. Quotes from Francophone participants were translated into English by the research team after data analysis was completed.

### Journey of Inuit Residents Into the Program

**Figure 2.**
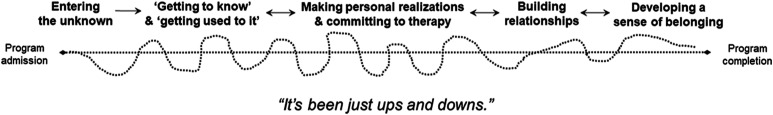
Journey of Inuit participants into the centre’s programs.

Most residents followed similar stages during their journey into the program (see Figure 2); each journey being influenced by their many encounters with other residents and staff. All residents we met, Inuit and non-Inuit alike, experienced some form of shock in the beginning of their program, with the first days or weeks being very unsettling. For most Inuit participants, the program structure and environment were very different from what they knew and were described as challenging to adjust to. At first, they felt controlled having to follow tight daily schedules and being subjected to the scrutiny of others. This feeling of restricted autonomy was amplified by having to stay in the same rooms for most of the day with limited outings beyond the centre’s premises.

For Inuit participants, working with “addicts” who “need fixing themselves” (in their words) was especially upsetting at first. Many Inuit needed two to three months – representing a third to a half of their program – before starting to settle down and to use the proposed therapeutic tools. “Getting to know” the place, the people and the program was a key process for Inuit participants to feel more comfortable and safer in their new environment.

“First came here, I felt very insecure. Because I didn’t know what’s going on, I didn’t know the people. But then I got to know them. I felt more secure.” (Elisapie)

Inuit participants who stayed in the program eventually got “used to it” and came to better understand and appreciate the program. Along their journey, they also made personal realizations that led them to commit more to the program. By interacting more with the other residents and staff using the program tools, Inuit participants developed new relationships based on having experienced similar life situations and emotions. When further developed and sustained over time, such connections eventually led Inuit participants to feel that they belonged to the ‘family’ or ‘community’ of program residents.

“As far as I’m concerned, this is my second family.” (Mae)

While the above provides a general overview of a ‘typical’ journey in the program, it should not be understood as the journey of all Inuit participants, nor as a linear one. The stories we heard highlight that all residents had ups and downs at all stages of the program, yet all experiences were unique. Not all residents we met completed their program, and some had returned for another program, regardless of whether or not they had completed the previous one.

### Navigating the Program Between Contrasting, Interrelated Situations and Feelings

#### Having Inuit Peers

“First time when I arrived, I saw [name of a particular Inuit resident]. I was all safe.” (Annie)

Having Inuit peers, especially when first arriving in the program, was valuable to most Inuit participants as it helped them to be more at ease in this unfamiliar environment. In addition to offering the opportunity to speak in their first language, Inuit participants reported that they were more open to talk to Inuit peers and felt better understood by them given their common understanding of Inuit historical experience and present situation. In contrast, conversations with non-Inuit were sometimes described as “awkward” due to language barriers or having different sociocultural referents.

In the same way, Inuit participants were greatly appreciative of and touched by the occasional visits by Inuit Elders, and deplored the comments they received every now and again from some non-Inuit residents who seemed to see this activity as a privilege and/or did not recognize its therapeutic value. Among other things, these meetings with Elders and other Inuit residents provided them with meaningful support and motivation to continue their healing journey.

“[When we meet with Elders], they think we’re getting special treatment. I try to explain to some of the girls that you know, we do our therapy too, it’s not like we’re just going out to have fun and eat country food. We have our own little therapeutic community too.” (Minnie)

Having Inuit peers was also important to Inuit participants because it helped them to develop relationships with fellow Inuit while sober. Nevertheless, these relationships were sometimes conflictual, as some Inuit participants reported being “put down” by Inuit peers at times, a situation that resulted in acute feelings of loneliness and isolation.

The presence of Inuit residents in programs fluctuated over time, depending on demand and available space. There were periods when there was only one Inuit resident and others when there were several (up to eight) or none. Some reported feeling alone and isolated when they were the lone Inuk in their program, whereas some others, while they also found the situation difficult, nevertheless saw it as an opportunity to connect with non-Inuit. In all cases, Inuit participants were concerned when leaving the program meant there would be a single Inuk remaining.

From the staff, there was a clear recognition of the importance and benefits of having more than one Inuk at a time in the program to facilitate their integration. However, some staff questioned the depth or quality of the relationship between Inuit residents, particularly when they came from the same northern village or were related otherwise, with some of these relationships in the program being seen as mostly based on convenience.

#### Having Limitations Imposed on One’s Ways of Being and Doing

At different points in their program, Inuit participants felt forced to do things they did not want to or that they did not understand. Likewise, some reported they felt prevented from acting according to their personal and/or collective ways of being and doing:

“It was very difficult for me at first because I was told… I don’t know how many times, not to be around Inuit, as much. [...] It was very difficult for me. I can’t just seem to… not be around them, because I’m so used to them. Like even though I didn’t know them for, like, a long time. Like if there’s Inuit, I’ll go to them.” (Maggie)

In some situations, Inuit participants felt they were treated differently specifically because they were Inuit, such as when they were asked to stay away from fellow Inuit and to mix more with other residents or when they were asked not to speak in Inuktitut so everybody could understand their conversations. When experienced or perceived by Inuit residents as manifestations of racism, these situations impinged on their experience of cultural safety in the program:

“In the beginning, I wasn't hanging out with the French girls or English girls or... either talking to them. I was just talking to [name of a given Inuit resident] and the other Inuit girls, mostly. And they would tell us, like ‘you Inuit girls’, ‘you Inuit girls’... And… I just felt like they were racist. And that they wanted us differently than the other girls. [...] And, I feel, I just felt watched.” (Elisapie)

Staff rather explained that they would intervene whenever they perceived that a “negative alliance” or an “unhealthy dynamic” existed between residents or group of residents in order to preserve the safety aspect of community dynamics and the integrity of the therapeutic approach. Although this course of action was not specific to Inuit residents, it was said to be likely happening with them because of their inclination to group together, sometimes without letting other people into their circle.

In situations where Inuit were asked to stay apart from each other, they would usually come to see benefits in that they were able to develop relationships with at least some non-Inuit residents; however, if the same intervention was made too early in their program or at a time when they had not yet developed a basic relationship with at least one other resident in the program, Inuit participants felt isolated and not listened to.

In contrast to the community dynamics that made them feel different or controlled, Inuit participants appreciated being able to have some input in their own individual plans of care in terms of what they felt they needed to improve instead of being told what to do, as this made them feel that their view matters.

#### Facing Ignorance and Misperceptions

“They only understand I live in a violent home. In a cold [climate]. With animals. *[laughing]* And they understand that I live in a lot of places where there is alcohol and drugs.” (Louisa)

For Inuit participants, having to live and work with non-Inuit was uncomfortable at times due to their ignorance of, and misperceptions about, the northern/Inuit context. From the Inuit participants’ perspective, non-Inuit residents and staff had a very limited knowledge and understanding of Inuit history and ways, the kind of life Inuit go through every day, and resources available in their communities. Staff acknowledged this perception describing how judgemental and reluctant they were at first to change their ways. Some staff have had (or still had) concerns over some Inuit customary practices. This sometimes led them to try to change Inuit residents’ ways, including how they take care of their loved ones or spend their money, even when it was not related to the reason for their participation in the program.

Throughout their program, Inuit participants faced many situations where the ignorance or misperceptions of non-Inuit residents and staff translated into negative experiences for them. As an example, staff participants shared their perception over how the northern environment is challenging for anyone who struggles with alcohol or drug issues to stay sober. Combined with an absence of knowledge of the resources available in Inuit communities, this concern led some staff – and non-Inuit residents as well – to adopt an overprotective approach towards Inuit residents and to suggest that they should consider not returning home after their program. In this view, there was little recognition of how wrenching a choice between home and sobriety could be, nor of the challenges Inuit would likely face by moving to a southern city (e.g. living without one’s relatives around and finding an apartment and a job). Inuit participants were hurt whenever staff and non-Inuit residents verbalized or acted as if they were thinking that all Inuit will relapse at the end of their program and/or they did not believe in their ability to stay sober in their home community. They felt mistrusted in such situations, which resulted in strong feelings of sadness, bitterness and anger. Inuit participants also felt uncomfortable, different or judged in situations where others were reacting to or asking them to change their ways, as described above. These negative experiences were usually associated with Inuit closing themselves off to others and/or to the program.

#### Having Conversations and Dialogue

Many of the challenging situations faced by Inuit participants sparked conversations and open dialogue among staff and residents, which helped to reduce tensions and to foster openness and trust. Staff participants reported the need to better understand Inuit life context and culture to be able to adjust their interventions accordingly, and they view interactions with Inuit residents as opportunities for non-Inuit to create relationships with Inuit while learning more about them individually and collectively. In that respect, staff often encouraged Inuit residents to talk about where they come from to non-Inuit residents and staff. Some Inuit participants were more open and comfortable to do so than others, depending on their personal traits and life experiences, as well as the context and subject matter.

Dialogue did help, most of the time, although educating non-Inuit represented an additional burden on Inuit participants. One thing that helped to alleviate this burden was the fact that some staff members had begun to travel regularly to the North to offer aftercare support. For Inuit participants, this contributed to a sense of being better understood, and for the staff who had gained this work experience in the North, it made it easier to build connections with Inuit residents thereafter. These staff usually became ‘allies’ for Inuit participants as they had witnessed and could corroborate information regarding their living conditions and realities:

“I used to be so wishing that staff could go up North. Now they have after-care group. Now they understand more what we go through with up North. So that is really helping too.” (Jeannie)

#### Facing Language Barriers

Throughout their program, language barriers presented significant challenges for many Inuit participants. At the time of data collection, most Inuit were more comfortable speaking in Inuktitut than in English, and only a few were fluent in French. In contrast, most non-Inuit residents were Francophones and had limited proficiency of English. Most staff were fluent in French and English, but none spoke Inuktitut. For Inuit, being in such a place contributed to a lack of comfort, being closed off and loneliness. Some had the sense of not being in the right place, especially at the beginning of their program.

Language barriers created a gap among the residents because of limited verbal interaction and communication, which contributed to misunderstandings and hindered the development of relationships with non-Inuit residents. To some extent, the language barriers also impacted the understanding that Inuit participants had of the program structure, routines and therapeutic tools, as well as their retention, participation and/or progression in the program. Having to use English or French represented an additional challenge when they wanted to express themselves, particularly when it came to sharing their feelings. Some Inuit were afraid to be judged or laughed at for the way they express themselves in another language, whereas others were hard on themselves for being slow or remaining silent in their interactions with non-Inuit. As most everyday conversations were in French and not everything was automatically translated into English during group meetings, Inuit reported being annoyed or frustrated having to constantly ask other residents or staff for translation into English (or Inuktitut) when their need for translation was known by all. Conversely, the staff saw these interactions as opportunities to learn how to be assertive and take one’s place in a group. Inuit participants were greatly appreciative when translation was readily offered, including during celebrations and at times when decisions about the affairs of the resident community were being made. This gave them access to the information that was being shared and made them feel that they were considered as full members of the resident community.

Beyond these considerations, language and communication issues reflected the level of relational comfort with residents and staff. When they first came into the program, some Inuit participants were not comfortable enough to let people know that they understood or spoke some French or, in a few cases, that they were actually fluent in French. Part of this was explained by the fact that they have limited opportunities to learn and use French in the North and needed time to be more confident in their abilities to use it in their interactions with Francophones. This was also the case for English although there was greater familiarity and fluency. Language was nevertheless intertwined with closedness and mistrust. Some Inuit participants have hidden, sometimes for weeks or even months, the fact that they were understanding what was going on in French to protect their distance from others and the program because of trust issues and/or to resist to the program:

[Name of an Inuit resident] told me that he does “not trust White people” and that he does not speak to them. Although he studied French in school and understands it very well, he said that nobody knows about it in the program and he acts as if he always needs the translation. (excerpt from field notes)

Language issues usually receded over time as Inuit residents became more familiar with the people and the program structure.

#### Being in a Supportive and Caring Environment

“There’s a lot of care. […] I don’t see care… much at home, as much as they do here. It makes me… I’m part of the group. Just the feeling of the thing they care for me. And that they’re there for me. Just makes me I’m part of this, the community.” (Elisapie)

Ultimately, Inuit participants found support and care through the relationships they developed with other residents and staff. For Inuit participants, being cared for meant having people who were there for them, to support them. Such people were described as sober, honest, well-meaning, helpful, empathetic and caring. They were welcoming and inclusive, reaching out to them and sharing their life story with them. They were taking an interest in them, listening to them and showing consideration and understanding of their situation.

These attributes contributed to feelings of trust and safety among Inuit participants. Being cared for made Inuit participants “feel important”. It helped them to feel less lonely, increased their self-esteem and gave them a reason to be hopeful for the future. Many Inuit participants had gone through traumatic events and had relatives and friends who were less available to offer appropriate support, especially emotional support, because of their own drinking and/or drug problems. In turn, following their experience in the program, several Inuit participants have said they wanted to be a caring person for their loved ones and/or to support other Inuit who are struggling with addiction as they did.

**Figure 3.**
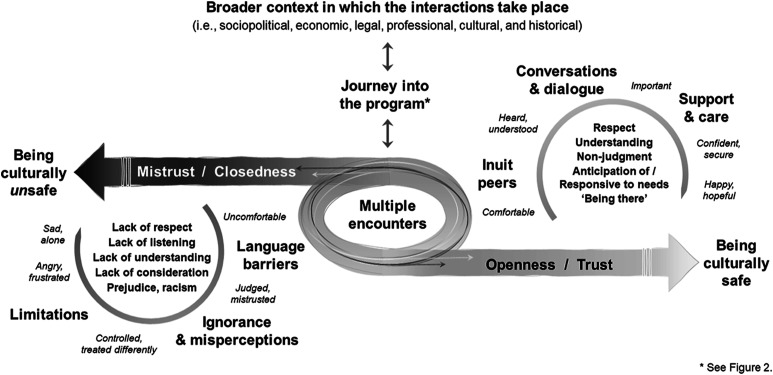
Relational interplay influencing the overall experience and cultural safety of Inuit participants in the centre’s programs.

From the overall experience of Inuit participants, we developed a model which proposes a way of understanding the relational interplay influencing their cultural safety in this setting (see Figure 3). The quality and outcome of the many encounters they had with the staff and other residents were influential in the development of trust throughout their program, which in turn was contributing to their feeling of being culturally safe. Some of these encounters were associated with uncomfortable or upsetting experiences related to facing or perceiving a lack of respect, listening, understanding and consideration for oneself or one’s life circumstances, as well as prejudice or racism (lower left circle). In contrast, other encounters were associated comforting and secure experiences related to interactions based on respect, understanding, non-judgement and care (upper right circle).

### Discussion

We examined the experiences of Inuit in a mainstream addiction rehabilitation centre to understand how cultural safety develops and unfolds in this context, using a case study design. The unique characteristics of the selected case allowed for a longitudinal examination of factors contributing to cultural safety within a complex intervention setting; in this case, long-term mainstream residential programs based on a therapeutic community approach. Our findings highlight that the relational dimension is central to understanding the Inuit experience of programs in a longitudinal perspective. The quality and outcome of the multiple encounters they had with the staff and other residents was a major influence on the development of trust and cultural safety over the course of their program. Key elements to promoting cultural safety in this context included having Inuit peers, having conversations and dialogue, and being in a supportive and caring environment, whereas having limitations imposed on one’s ways of being and doing, facing ignorance and misperceptions and facing language barriers were rather hindering cultural safety. Our study emphasizes the need to consider the dynamic interplay between contexts and practices that promote and hinder cultural safety, particularly in long-term care programs, to enhance the care experience of Inuit and likely of other population groups.

### Trust as a Necessary Condition for Cultural Safety

Historical and ongoing experiences of oppression and marginalization of Indigenous peoples resulted in a loss of trust in self, family/clan, community, and government and outsiders, all of which impacts the way Indigenous people engage in life, build relationships and participate in various activities, including treatment programs (Thibodeau & North Peigan, 2007). Like other Indigenous peoples, Inuit report negative experiences with health and social services, which they describe as “difficult to trust and potentially dangerous” (Fraser & Nadeau, 2015S. L. Fraser & Nadeau, 2015, p. 295). Nevertheless, trust is crucial for the therapeutic alliance and for group cohesion and functioning (von Greiff & Skogens, 2014; Yalom & Leszcz, 2005), including in therapeutic communities.

By emphasizing the central role of trust in fostering a sense of cultural safety in Inuit, our findings point to its dynamic nature as a relational process outcome. Trust needs to be established for “exotic differences” in the care relationship be exposed, discussed and negotiated (Ramsden, 2002, p. 179). Trust itself builds iteratively on the basis of social interactions over time and is “dynamic, volatile, and constantly renegotiated” (Carr, 2001, p. 35). In clinical contexts where vulnerability is unavoidable, the affective component of trust is prominent (M. A. Hall et al., 2001) and grounded in “relationships and affective bonds generated through interaction, empathy and identification with others” (Calnan & Rowe, 2007, p. 284). In our study, Inuit participants were more likely to be comfortable with Inuit peers at first and eventually expanded their zone of comfort after getting to know other residents and staff and developing relationships with them based on similar experiences and emotions. That Inuit participants eventually felt they were part of the residents’ ‘family’ or ‘community’ also suggests that they were able to develop interpersonal trust with at least a few people over their journey in the program. Developing a sense of belonging within a supportive and caring environment has been found to have a positive and validating effect on experiences of Indigenous people accessing preventive care (Van Herk et al., 2012).

Opportunities to develop trust are generally more limited in programs characterized by rigidity and restrictions imposed on its users, which may contribute to perpetuating mistrust of the system (Law et al., 2019). Collective and individual experiences of Inuit with institutions or programs characterized by power imbalances and surveillance modes that impinge upon or limit their autonomy – such as the residential school system and justice and child welfare systems where they are overrepresented – may have amplified their impression of being watched, judged or belittled when subjected to the scrutiny of others within the program, and contributed to their feeling of being unsafe.

### Quality of Communication as a Cornerstone for Cultural Safety

Language barriers and cross-cultural misunderstandings have been observed to negatively impact the self-esteem of Inuit when interacting with southern mainstream health services (Tester et al., 2001), which is likely to diminish their well-being (Woods, 2010). In our study, some Inuit participants were prone to self-blame for their personal failings when they were unable to express themselves well or were slow to react in either one of the languages in which the programs were offered. For them, communication was easier with Inuit peers due to common language and referents. As Inuktitut is a symbol of Inuit identity (Dorais, 1995), having the opportunity to express themselves in Inuktitut was important and being denied such occasions was unacceptable to them. A Norwegian study also found that neglecting the Sami language and not offering interpreting service in health care encounters were considered culturally unsafe by North Sami-speaking patients (Mehus et al., 2019). Besides language, situations where non-Inuit were reacting negatively to Inuit customary practices were sources of frustration and, depending on the situation and the person, resulted in withdrawal or resistance. In this context, the occasional activities dedicated to Inuit residents were valued as they gave them a break from intensive cross-cultural interactions. Dedicated, Indigenous-only time and space were highlighted by other authors likewise as being comfortable and culturally safe for Indigenous peoples (Firestone et al., 2019).

Providing culturally safe care implies being able not only to notice, recognize and interpret correctly various communication cues including verbal, body and gestural language and silence; but also being knowledgeable and reflexive about our own communication patterns (Møller, 2016). Not knowing or adjusting one’s ways of interacting may lead Inuit to the impression of a lack of consideration (Ruderman & Weller, 1981). In the absence of other people with Inuit cultural and linguistic skills, such as Inuit staff, Elders or interpreters, Inuit participants were often left with the role of cultural broker for non-Inuit residents and staff. There were times when other people, especially staff members with working experience in the North, validated what Inuit residents were saying and advocated on their behalf in situations likely to be prejudicial to them. The fact that these staff were in a social position that enables them to challenge authority (O'Neil, 1989) may partly explain why their words seemed to carry more weight than those of the Inuit residents who were primarily affected.

Communication is a medium through which power can be exercised (Jennings et al., 2018). In our study, ‘power over’ manifested in multiple ways through verbal and non-verbal interactions such as dismissing, othering, judging, making statements based on stereotypes and using a foreign or unfamiliar language without proper translation (Browne & Fiske, 2001; Jennings et al., 2018). Although such manifestations were not specific to the interactions from non-Inuit towards Inuit, they have the potential to perpetuate inequities between the two groups when used (even inadvertently) by non-Inuit in a way that excludes or limits the full participation of Inuit in program activities or their integration in the resident community. Power to disagree or resist was also present, which sometimes passively manifested by Inuit participants not paying attention to the message or ‘just saying yes’ when feeling too powerless to challenge a situation or what was asked from oneself (Jennings et al., 2018; Møller, 2005).

### The Need to Reflect on Power Dynamics

To the extent that the therapeutic community approach is essentially based on peer support, the relational dynamics are necessarily different from those in other contexts where a care provider has a status and knowledge that places him or her in an expert position relative to the care recipient. That said, therapeutic communities remain places where power issues are present, tied to the structure and program modalities that involve certain types of interactions among their members, residents and staff included. That all staff and most residents were non-Inuit also points to the need to explicitly consider the macro context influences on interactions and power dynamics (Fraser et al., 2015; O'Neil, 1989). This is all the more important in cognitively and emotionally challenging working/living environments since stressful conditions may lead to the activation of implicit racial/ethnic biases (i.e. prejudicial attitudes and/or stereotypes) from which unwitting discrimination can arise (W. J. Hall et al., 2015; van Ryn & Fu, 2003).

Most participants had a very limited awareness or understanding of the historical and socio-political context in which their interactions in the program took place. Raised and socialized in different yet interconnected social worlds, Inuit and non-Inuit inevitably have different embodied experiences of life and of the persistent legacies streaming from colonial history, which could lead to very different interpretations or actions in response to a same situation. In some cases, the pervasiveness of racism and discrimination may have played a role in Inuit participants’ lives, including in the very pathways that brought them into treatment at the centre. As most Inuit participants were women, it should also be noted that compared to Indigenous men, Indigenous women and girls are known to be disproportionately impacted by prejudice and discrimination and feel less safe in their interactions with the health care system due to the intersection of racism against Indigenous peoples, misogyny and gender discrimination (Turpel-Lafond, 2020). As for the perspectives of non-Inuit, they were likely influenced by their own experiences, if any, of working/living with Inuit in the program or in other particular situations (e.g. group home, detention and homelessness), as well as by dominant discourses about culture, egalitarianism and Indigenous peoples (Browne, 2005).

To foster a therapeutic community spirit, the staff focused on the commonalities in residents’ experiences. Although this strategy had some benefits, it also entails potential risks to cultural safety as it may result in situations where individuals’ experiences are being examined and understood only in the light of these commonalities (perceived or actual). For example, some staff were likely to interpret the fact that Inuit residents felt judged or misunderstood as a common trait of people who use alcohol/drugs, without necessarily considering the ways in which multiple systems of oppression such as racism, colonialism, classism and sexism intersect to structure the unique life experiences of each resident (Hole et al., 2015). For Inuit, not having the feeling that their social experience as a whole is heard, understood and considered may negatively influence the evaluation of their care encounters with non-Inuit staff and residents (O'Neil, 1989). As for staff, failure to consider the broader socio-political and historical contexts may lead them to interpret everyday clinical situations as reflective of personal opinions or sensitivities of Inuit residents; this would hinder the identification of strategies that may disrupt individual and institutional practices that, albeit unintentionally, may contribute to or perpetuating racialization and injustices (Browne, 2017).

### Implications for Practice

Although the responsibility of providing a culturally safe environment lies with the care providers and institutions, the actual outcome of their efforts also depends on the care recipients who have to engage themselves in the relational care process to actualize cultural safety. For managers and care providers serving Inuit, it is essential to understand and consider the gap in trust and to create the conditions for mutual trust to develop. A closer attention to communication issues and power imbalances would also be warranted. A better understanding of the immediate and broader contexts in which care is provided could help moving forward in this direction. Finally, care providers and institutions can refer to equity-oriented initiatives and tools to foster the critical reflexivity and organizational changes necessary to create culturally safe care environments (e.g. Browne et al., 2018; Jones, 2018; Nixon, 2019; Plamondon, 2020).

### Study Limitations

This study contributes to the understanding of key features of culturally safe addiction rehabilitation programs for Inuit, especially women. Further research with Inuit men is warranted as their care experiences might be different. Our observations are also intrinsically linked to the therapeutic context and approach in the participating centre. During data analysis, it was difficult at times to distinguish the relational dynamics associated with cultural safety from the recovery process in group therapy which involves self-identification or self-affiliation to the group. The transferability of study findings to other settings, especially where programs are shorter and/or focusing on individual counselling, would need further exploration. Finally, we cannot completely avoid the risk of essentialization in the way we represented Inuit and non-Inuit participants in the study, although we have tried to minimize distortions by using reflexivity and perspective throughout the research process.

## Conclusion

Our study reinforces the idea that being culturally safe relates to developing trustful relationships with the people around oneself. In a place that is likely to pose a cultural risk due to a marked sociocultural distance and power differentials, some encounters or situations would foster a sense of cultural safety while others will go the other way, depending on people, context and time. Consequently, we find that cultural safety cannot be conceptualized as a simple dichotomous outcome of care nor as a consistent one, especially in a complex long-term intervention.
